# Locomotor Development Prediction Based on Statistical Model Parameters Identification

**DOI:** 10.1155/2012/548208

**Published:** 2012-12-10

**Authors:** A. V. Wildemann, A. A. Tashkinov, V. A. Bronnikov

**Affiliations:** ^1^The Center of Mathematical Modelling of Medicosocial Systems and Processes, The State National Research Politechnical University of Perm, Perm 614990, Russia; ^2^The Department of Physical Culture and Health, The Perm State Medical Academy, Perm 614000, Russia

## Abstract

This paper introduces an approach for parameters identification of a statistical predicting model with the use of the available individual data. Unknown parameters are separated into two groups: the ones specifying the average trend over large set of individuals and the ones describing the details of a concrete person. In order to calculate the vector of unknown parameters, a multidimensional constrained optimization problem is solved minimizing the discrepancy between real data and the model prediction over the set of feasible solutions. Both the individual retrospective data and factors influencing the individual dynamics are taken into account. The application of the method for predicting the movement of a patient with congenital motility disorders is considered.

## 1. Introduction

Multidimensional statistics methods, including predicting and dependencies analysis, are required to research complex systems with many random factors [1–4]. In order to obtain more reliable individual prediction, the patient specific information needs to be taken into account to correct statistical model parameters. 

Modeling of biomedical systems provides actual application of mathematical methods and algorithms. This paper studies the problem of locomotor development prediction for people with congenital motility abnormalities. The authors propose an approach to identify the parameters of a statistical predictive model taking into account the available set of individual data.

## 2. Current State 

Mathematical methods and information technologies are important tools in modern biomedical research. Together with physicomechanical modeling of physiological systems [5–7], the study of statistical properties of disease courses for patients with similar diagnoses is also of certain interest. Statistical predictive modeling in medicine covers a wide range of areas including cardiology [8–10], pulmonology [11–13], neurology [14, 15], and others. 

One of the disadvantages of statistical predictive models is that they are only applicable to an average individual. In order to get a more accurate prediction it is necessary to identify statistical predictive model parameters using data for a concrete person. Using this approach which is known as “individual prediction” method [16, 17] in medical science is of major interest for the modern interdisciplinary scientific development.

## 3. Materials and Methods 

### 3.1. Initial Data Analysis

The motility index *Y* quantifies the level of locomotor development. In order to calculate *Y* an expert evaluates 12 different groups of locomotor skills. The expert estimations are then arranged according to the five-point scale and the index *Y* is defined as the sum of the estimations. Due to the construction algorithm the index *Y* is defined within the interval [0,60]. The best locomotor development corresponds to the top boundary of parameter *Y* and the worst locomotor development corresponds to the lowest value.

The motility index dependence on age for a group of patients is considered to be a random process *Y*(*t*), where *t* is the patient's age. The process *y* ^(*J*)^(*t*) corresponds to each patient *J* in the group.

To analyze the stochastic process structure the following correlation function is used as follows:

(1) $$\begin{array}{c} r_{y}\left(t,t^{\prime }\right)=\frac{M\left[\overset{{}^{\circ}}{Y}\left(t\right)\overset{{}^{\circ}}{Y}\left(t^{\prime }\right)\right]}{\sqrt{D_{y}\left(t\right)D_{y}\left(t^{\prime }\right)}}, \end{array}$$

where $\overset{{}^{\circ}}{Y}(t)$ and $\overset{{}^{\circ}}{Y}\left(t^{\prime }\right)$ are time slices of the centred random process $\overset{{}^{\circ}}{Y}(t)$ at the time *t* and *t*′, *D*_*y*_(*t*) and *D*_*y*_(*t*′) are dispersions of *Y*(*t*) at the moments *t* and *t*′, and *M* is the expectation operator.

The available statistics database contains 157 observations of cerebral palsy patients. The research sample was based on occasional patient visits to a rehabilitation centre during their first nine years of life. Experts determined each patient's motility index during their visits. Detailed information was collected on each patient: medical status of parents and close relatives prior to the individual's conception and characteristics of prenatal and perinatal periods (prenatal and intranatal factors).

Despite the presence of relatively large number of observations, it was only possible to focus on 5 people who were constantly observed during the entire research period. Given the data limitation the study of locomotor skills development was conducted on a group of 5 people (*N* = 5) and the complete initial sample of 157 observations was used for calculating the average parameters.

**Figure 1:**
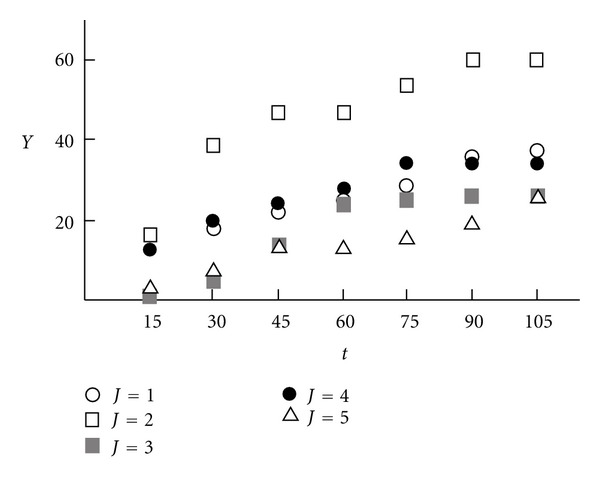
The realization of motility index.

**Figure 2:**
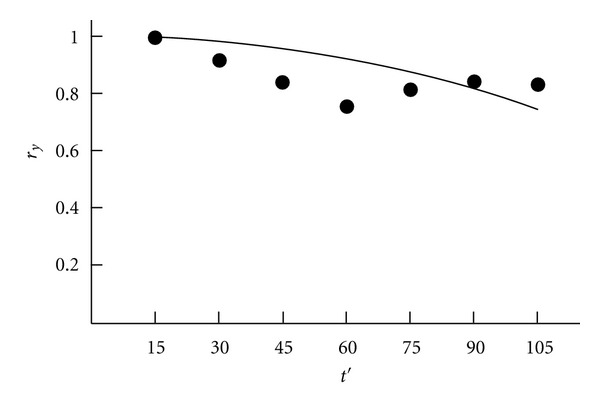
The correlation function of motility index.

A scatter diagram of random process *Y*(*t*) is shown in Figure 1, where *t* is the age (in months).  Figure 2 shows the graph of the corresponding correlation function for fixed age *t* = 15 (months).

When the correlation function is close to one, the process is characterized by a strong dependence between the time slices. This indicates that the process realizations are similar. The similarity is the necessary condition for the application of the proposed method.

### 3.2. General Algorithm for Individual Prediction

Individual prediction of motility index is based on the following algorithm. 

The first step determines the age dependence of the average motility. The consecutive selection method of the exponential terms [16] is used for this purpose. According to this method, the average motility index trend is represented as follows:

(2) $$\begin{array}{c} f\left(t\right)=a_{0}+\sum_{q=1}^{Q}a_{q}\left(1-e^{-\lambda_{q}t}\right), \end{array}$$

which includes the following unknown parameters: $a_{0},\;a_{q},\;\lambda_{q}(q=\bar{1,Q})$.

The individual motility index trend is constructed as bounded from above monotonically increasing function:

(3) $$\begin{array}{c} F^{\left(J\right)}\left(t\right)=A_{0}^{\left(J\right)}+\sum_{q=1}^{Q}A_{q}^{\left(J\right)}\left(1-e^{-\lambda_{q}t}\right). \end{array}$$

This paper studies the locomotor skills accumulation process only. The loss of such skills is not taken into account therefore the monotonically increasing assumption could be used. The motility index boundaries are defined by their calculation method (from 0 to 60).

The generalized factor of prenatal and intranatal conditions is introduced as follows:

(4) $$\begin{array}{c} \Phi =\sum_{r}\delta_{r}P_{r}, \end{array}$$

where *P*_*r*_ is the risk factors during pregnancy and birth that have the highest influence on the motility index, and *δ*_*r*_ is the correlation between *P*_*r*_ and the motility index.

The assumption of *P*_*r*_ factors being responsible for development delay and, as such, affecting the absolute term value *A*_0_^(*J*)^ is used.

The motility index value *A*_0_^(*J*)^|_Φ=0_ at time 0 with no prenatal and intranatal risk factors taken into account is calculated by solving the following convex optimization problem with constraints in the form of inequalities. 

Find ${{\tilde{A}}_{0}^{\left(J\right)}|}_{\Phi =0}$ and ${\tilde{A}}_{q}^{\left(J\right)}(q=\bar{1,Q})$ such that:

(5) $$\begin{array}{c} \sum_{\gamma =1}^{\Theta_{0}}{\left({A_{0}^{\left(J\right)}|}_{\Phi =0}+\sum_{q=1}^{Q}A_{q}^{\left(J\right)}\left(1-e^{-\lambda_{q}t_{\gamma }}\right)-y_{\gamma }^{\left(J\right)}\right)}^{2}\to \min,\\ A_{q}^{\left(J\right)}\geq 0\left(q=\bar{1,Q}\right),\\ {A_{0}^{\left(J\right)}|}_{\Phi =0}+\sum_{q=1}^{Q}A_{q}^{\left(J\right)}\left(1-e^{-\lambda_{q}t_{\Theta }}\right)\leq y_{\max}, \end{array}$$

where $\lambda_{q}(q=\bar{1,Q}$) are known values calculated during the average trend *f*(*t*) identification, Θ_0_ is the number of real motility index values in the interval of the initial individual observation *τ*_0_, *y*_*γ*_^(*J*)^ is the *J*'s patient real motility index value at the moment of time *t*_*γ*_ ∈ *τ*_0_, *t*_Θ_ is the prediction horizon, and *y*_max⁡_ is the possible highest value of motility index.

The first expression in system (5) is the minimum condition for the sum of square deviations from real motility index values. The second expression is the motility monotonic growth condition. The third one is the motility index restriction condition.

The problem (5) could either be solved numerically or analytically via the Kuhn-Tucker theorem [18].

The angular coefficient *μ* (which determines how strong the *P*_*r*_ factors influence the initial motility index changes) is calculated using linear approximation of the real motility index dependence at the initial time on the generalized prenatal and intranatal factors for a group of patients. The calculated coefficient *μ* is used for *A*_0_^(*J*)^ adjustment taking the influence of prenatal and intranatal factors into account: *A*_0_^(*J*)^ = *A*_0_^(*J*)^|_Φ=0_ + *μ*Φ^(*J*)^. The other individual coefficients $A_{q}^{\left(J\right)}(q=\bar{1,Q})$ are calculated by solving the optimization problem (5) with the adjusted coefficient *A*_0_^(*J*)^ obtained during the previous step.

## 4. Results and Discussion

The practical applicability of this method is illustrated for a patient J*. The person J* is a cerebral palsy patient and was observed at the Perm Center of Complex Rehabilitation for People with Disabilities during the first nine years of life.

The entire observation time period is divided into two intervals: base period (first four years) and prediction period (next five years). The first period data is used to develop the motility index prediction model while the second period data serve as a test for prediction results (Table 1).

**Figure 3:**
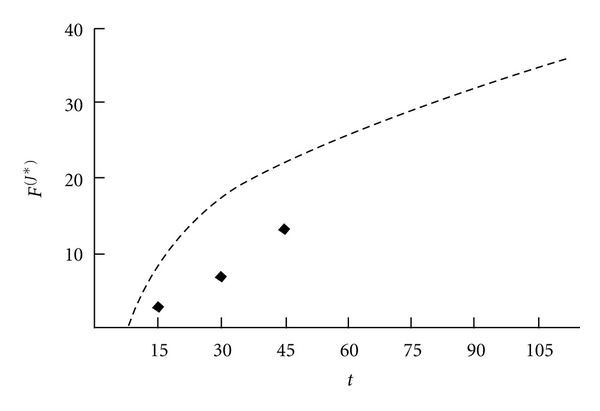
The individual data (black dots) and the average trend (dashed line) of motility index.

The initial locomotor development dynamics of the observed patient (black dots in Figure 3) differ significantly from the age dependence of average motility index for a group of patients with similar diagnosis (dotted line in Figure 3). Therefore the prediction based on the average trend only will not produce acceptable accuracy in final results. At the same time the prediction based on the individual data only will also not be accurate since the base period is shorter than the prediction period. The proposed method which corrects the average trend parameters using individual patient data is further carried out to increase the prediction accuracy.

Using the method of sequential selection of exponential terms [16] results in the expression for the average motility index trend:

(6) $$\begin{array}{c} f\left(t\right)=-16.49+66.87\left(1-e^{-0.01t}\right)+28.39\left(1-e^{-0.09t}\right). \end{array}$$

The correlation analysis method is applied to identify a set of variables that are further used as prenatal and intranatal conditions (Table 2) that significantly influence the motility index for a group of patients with cerebral palsy. The sample of 157 observations was used to calculate the correlation coefficient [17].

The individual values of prenatal and intranatal factors are shown in Table 3: “0” means “no”, “1” means “yes”.

The generalized factor of prenatal and intranatal conditions with the individual data and the correlations shown in Tables 2 and 3 being taken into account has the value Φ^(J*)^ = 0.67.

Solving the system (5) gives the motility index at the initial time in the absence of prenatal and intranatal risk factors: *A*_0_^(J*)^|_Φ=0_ = −3.00.

The coefficient *μ* obtained by the least-squares method [19] for the group of patients is −16.68. The corresponding adjusted initial motility index is calculated as: *A*_0_^(J*)^ = *A*_0_^(J*)^|_Φ=0_ + *μ*Φ^(J*)^ = −14.17. The remaining coefficients are *A*_1_^(J*)^ = 60.52, *A*_2_^(J*)^ = 17.04.

**Figure 4:**
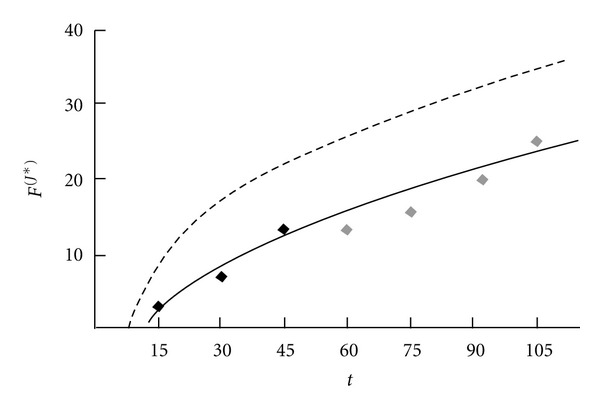
The control data (gray dots) and individual prediction (solid line) of motility index.

The identified individual motility index trend *F* ^(J*)^(*t*) = −14.17 + 60.52(1 − *e* ^−0.01*t*^) + 17.04(1 − *e* ^−0.09*t*^) is shown in Figure 4 (solid line). Compared to the average trend the individual predictive model is much closer to the control data (gray dots). The determination coefficient is *R* ^2^ = 0.92.

## 5. Conclusions

The proposed method of parameters identification for a statistical predictive model based on data set of patient information can be successfully applied conditioned on the correlation function being close to one. This method is based on multidimensional statistical analyses [1–4] and individual prediction approach [16, 17]. 

The application of the method to statistical modeling of medical and social systems resulted in an algorithm which does not require modeling of physical and mechanical processes. The obtained algorithm is based on statistical analysis only. The application of the algorithm significantly improves the prediction accuracy compared to the predictions based on average statistics. The improvement has been successfully demonstrated in an example of individual locomotor development prediction for a patient with congenital motility disorders.

The results show the advantages of the proposed method when the predicted index has a significant variation within the group with the prediction interval being larger than the base period.

## Figures and Tables

**Table 1:** The individual data of motility index.

	Prediction base period			Prediction period			
*t*_*γ*_, month	15	30	45	60	75	90	105
*y*_*γ*_^(J*)^	3.00	7.00	13.25	13.25	15.50	19.25	25.00

**Table 2:** The set of most significant prenatal and intranatal factors.

*P*_*r*_	The factors	*δ*_*r*_
*P*_1_	Signs of intrauterine infection	−0.50
*P*_2_	Signs of fetal hypoxia	−0.39
*P*_3_	Extremely low weight at birth	−0.28

**Table 3:** The individual data of prenatal and intranatal factors.

*P*_1_^(J*)^	*P*_2_^(J*)^	*P*_3_^(J*)^
0	1	1
